# Supplementary material for: Deception is associated with reduced social connection
Source: Commun Psychol. 2023 Sep 14;1:19. doi: 10.1038/s44271-023-00021-0 (PMC11041808; doi:10.1038/s44271-023-00021-0)
Supplement: Supplementary file 2 — Reporting Summary [file 44271_2023_21_MOESM2_ESM.pdf]

nature portfolio

Corresponding author(s): Leanne ten Brinke, Ph.D.

Last updated by author(s): Aug 9, 2023

Reporting Summary

Nature Portfolio wishes to improve the reproducibility of the work that we publish. This form provides structure for consistency and transparency in reporting. For further information on Nature Portfolio policies, see our Editorial Policies and the Editorial Policy Checklist.

Statistics

For all statistical analyses, confirm that the following items are present in the figure legend, table legend, main text, or Methods section.

n/a	Confirmed	
☐	☒	The exact sample size (n) for each experimental group/condition, given as a discrete number and unit of measurement
☐	☒	A statement on whether measurements were taken from distinct samples or whether the same sample was measured repeatedly
☐	☒	The statistical test(s) used AND whether they are one- or two-sided<br>*Only common tests should be described solely by name; describe more complex techniques in the Methods section.*
☐	☒	A description of all covariates tested
☐	☒	A description of any assumptions or corrections, such as tests of normality and adjustment for multiple comparisons
☐	☒	A full description of the statistical parameters including central tendency (e.g. means) or other basic estimates (e.g. regression coefficient) AND variation (e.g. standard deviation) or associated estimates of uncertainty (e.g. confidence intervals)
☐	☒	For null hypothesis testing, the test statistic (e.g. F, t, r) with confidence intervals, effect sizes, degrees of freedom and P value noted<br>*Give P values as exact values whenever suitable.*
☒	☐	For Bayesian analysis, information on the choice of priors and Markov chain Monte Carlo settings
☐	☒	For hierarchical and complex designs, identification of the appropriate level for tests and full reporting of outcomes
☐	☒	Estimates of effect sizes (e.g. Cohen's d, Pearson's r), indicating how they were calculated

Our web collection on statistics for biologists contains articles on many of the points above.

Software and code

Policy information about availability of computer code

Data collection	Study 1 is an analysis of open-source data, provided by BetterUp Inc. and detailed in Reece et al., 2022. Study 2 used Qualtrics with Chatplat integration to gather data and facilitate conversations, respectively. Study 3 used Qualtrics for data collection.
Data analysis	Data analysis was conducted using R (lmerTest package) and SPSS 27 with MLmed and PROCESS macros to support mediation models.

For manuscripts utilizing custom algorithms or software that are central to the research but not yet described in published literature, software must be made available to editors and reviewers. We strongly encourage code deposition in a community repository (e.g. GitHub). See the Nature Portfolio guidelines for submitting code & software for further information.

Data

Policy information about availability of data

All manuscripts must include a data availability statement. This statement should provide the following information, where applicable:

- Accession codes, unique identifiers, or web links for publicly available datasets
- A description of any restrictions on data availability
- For clinical datasets or third party data, please ensure that the statement adheres to our policy

Data used in Study 1 analysis is available for download from BetterUp Inc. here: https://betterup-data-requests.herokuapp.com/. Deidentified data for Studies 2 and 3 are available on OSF: https://osf.io/ezn7p/.

Human research participants

Policy information about studies involving human research participants and Sex and Gender in Research.

Reporting on sex and gender	We report self-identified gender information in the Participants section of all studies. Becuase we had no theoretical reason to expect gender (or sex) differences, no analyses included these variables. However, this data is available in the BetterUp Inc. CANDOR dataset (Study 1) and on OSF (Study 2 & 3) for researchers who may be interested in this data.
Population characteristics	Age and gender data, as well as how participants were recruited is provided in the Participants section of each study. None of these variables were includes as covariates in analyses.
Recruitment	Participants were recruited on Prolific for all studies. This recruitment method improves on common undergraduate samples, by including a greater diversity of age, SES, and race/ethnicity; however, it is still subject to self-section biases.
Ethics oversight	Study 1 was approved by Ethical & Independent Review Services. Studies 2 and 3 were approved by the University of British Columbia, Okanagan Behavioural Research Ethics Board.

Note that full information on the approval of the study protocol must also be provided in the manuscript.

Field-specific reporting

Please select the one below that is the best fit for your research. If you are not sure, read the appropriate sections before making your selection.

☐ Life sciences ☒ Behavioural & social sciences ☐ Ecological, evolutionary & environmental sciences

For a reference copy of the document with all sections, see nature.com/documents/nr-reporting-summary-flat.pdf

Behavioural & social sciences study design

All studies must disclose on these points even when the disclosure is negative.

Study description	All studies are quantitative in nature. Studies 1 and 3 use correlational designs, while Study 2 is experimental.
Research sample	The sample in Study 1 was recruited by BetterUp Inc. to create the CANDOR database. Recruitment on Prolific targeted individuals based in the United States and 18 years of age or older. Dyads were included in analyses if both participants completed the post-conversation ratings of personal trustworthiness, perceived trustworthiness of their partner, and the measure of interpersonal closeness with their partner. No further data exclusions were made. This resulted in a total of 388 complete dyads in the analyses below. Of these 776 participants, 423 identified as females, 321 as males, and 5 as other or prefer not to answer. Participants were an average age of 33.81 (SD = 10.98; range = 19 - 63). Twenty-seven participants did not provide age information.<br><br>The sample in Study 2 was recruited on Prolific, and was limited to individuals based in the US and 18 years of age or older. A total of 212 dyads provided complete data. Four dyads were removed prior to analysis for not following instructions (i.e., did not use conversation-starter questions). The final dataset included 208 dyads or N = 416 participants (189 men; 216 women; 8 non-binary; 3 gender not listed; 2 prefer not to say). The mean age of our participants was 37.92 (SD = 13.29; range: 18-84).<br><br>The sample in Study 3 was recruited on Prolific, and was limited to individuals based in the US and 18 years of age or older. N = 399 participants completed the study, and no data exclusions were made. Of these individuals, 178 participants identified as men, 206 as women, 11 as non-binary, and 4 selected 'prefer not to say'. One participant selected both 'woman' and 'non-binary'.
Sampling strategy	Study 1. We used all dyads with complete data that were available in the BetterUp Inc. CANDOR database.<br>Study 2. Our sample size goal (N = 200 dyads) was identified on the basis of similar research, involving stranger dyads in a chat-based, two-group experimental design (Huang et al., 2017; Study 1 & 2A).<br>Study 3. A power analysis indicated that N = 395 participants would be necessary to find a small effect (f2 = .02) in a multiple regression with three predictors, setting p = .05 and 1-β = 80%.
Data collection	Study 1 is an analysis of open-source data, provided by BetterUp Inc. and detailed in Reece et al., 2022. Conversations were faciliated and recorded using TokBox OpenTok Video API and survey responses were collected using Qualtrics. Study 2 used Qualtrics with Chatplat integration to gather data and facilitate conversations, respectively. Study 3 used Qualtrics for data collection.
Timing	Study 1. Between January and November 2020, six rounds of data collection yielded a total of 1656 dyadic conversations that were recorded over video chat.<br>Study 2. Data was collected between April 11 and May 25, 2022.<br>Study 3. Data was collected on October 13, 2022.
Data exclusions	Study 1. Dyads were excluded if they did not have complete data for the variables of interest.<br>Study 2. Dyads were excluded if they did not provide complete data, or if they did not follow study instructions.<br>Study 3. No data exclusions were made.

Non-participation	Participants who did not complete were presumed to have withdrawn consent; their data has been deleted.
Randomization	Only Study 2 was experimental in nature; random assignment was used to assign participants to roles and conditions.

Reporting for specific materials, systems and methods

We require information from authors about some types of materials, experimental systems and methods used in many studies. Here, indicate whether each material, system or method listed is relevant to your study. If you are not sure if a list item applies to your research, read the appropriate section before selecting a response.

Materials & experimental systems

n/a	Involved in the study	
☒	☐	Antibodies
☒	☐	Eukaryotic cell lines
☒	☐	Palaeontology and archaeology
☒	☐	Animals and other organisms
☒	☐	Clinical data
☒	☐	Dual use research of concern

Methods

n/a	Involved in the study	
☒	☐	ChIP-seq
☒	☐	Flow cytometry
☒	☐	MRI-based neuroimaging
